# Supplementary material for: Verification of AKT and CDK5 Gene and RNA Interference Combined with Irradiation to Mediate Fertility Changes in Plutella xylostella (Linnaeus)
Source: Int J Mol Sci. 2024 Apr 24;25(9):4623. doi: 10.3390/ijms25094623 (PMC11082963; doi:10.3390/ijms25094623)
Supplement: Supplementary file 1 [file ijms-25-04623-s001.zip › ijms-2941540-supplementary.pdf]

Table S1. Primers sequence used in this experiment.

Use of primers	Gene	Primer sequence (5′-3′)
qPCR primer	*PxPI3K*	F: CACGCCTCAGCAGCACATCG<br>R: CTCCTCTTGACCGCACACCTTG
	PxJAK2	F: GCCGCCAGATTACGAGAGGATTG<br>R: GGAAGCCGACGATTCAATGAGAGG
	PxmTOR	F: CGACACAAGGACAGCGTCATGG<br>R: GTAGAGGCGAGGTGGAGGGTTC
	PxPTEN	F: GAGACTGACCTGGCTGAAGACAAC<br>R: TGTGTTCCGTGGGCGTTTGAG
	PxAKT	F: GCCGAACCACCAAGACCTTCTG<br>R: GCCGCAGCACATCTCGTACAG
	PxPDK	F: CACTGCCCAAGACTACATT<br>R: CCTCCTTTTCTCGTTTTAT
	PxCDK5	F: CATGTGGTCCGCTGGCTGTATC<br>R: CGGCTTGTAGTCAGGCAGTTGG
	PxNFkB	F: GAGCATCACGGAGCAGCCATG<br>R: GGTATGTCTTGGTGCGGTTACTGG
	PxIKKα	F: ACTGCTGGAGAAATGGTT<br>R: ATCACTTTGCTTGCCTCT
dsRNA primer	T7 - *PxPI3K*	F: TAATACGACTCACTATAGGGGCCGGCTGGTCGAGGTGGTA<br>R: TAATACGACTCACTATAGGGCAAAGTCTTTGCTCAGTCGC
	T7 - *PxJAK2*	F: TAATACGACTCACTATAGGGAACGAAGCGACCATCACACT<br>R: TAATACGACTCACTATAGGGTTTGGACAAGGTGGATCGGG
	T7 - *PxmTOR*	F: TAATACGACTCACTATAGGGACACGAGGACTTGCGACAAG<br>R: TAATACGACTCACTATAGGGGGGATCTTCTCGGGGAACTT
	T7 – *PxPTEN*	F: TAATACGACTCACTATAGGGTGGTGCTGAGCCCCGCGCCC<br>R: TAATACGACTCACTATAGGGAGGGGCGCTCACCCGAGTCC
	T7 - *PxAKT*	F: TAATACGACTCACTATAGGGGCGGGTGGAGGCGCCCGGGT<br>R: TAATACGACTCACTATAGGGCATGGCATACAGCTTGCCCG
	T7 - *PxNFkB*	F: TAATACGACTCACTATAGGGATGGCTGATGTGGAATGCGA<br>R: TAATACGACTCACTATAGGGTAGCTCTGGGAGGTACGCTG
	T7 - *PxCDK5*	F: TAATACGACTCACTATAGGGTGAAGAAATACTTTGATAGT<br>R: TAATACGACTCACTATAGGGTGCCATGGCATCGTCAGCTG
	T7 - *PxPDK1*	F: TAATACGACTCACTATAGGGCACCATCCCCAACAAGTCCA<br>R: TAATACGACTCACTATAGGGACGGTGGAAGACCCGAAATC
	T7 - *PxIKKα*	F: TAATACGACTCACTATAGGGAGAACCGATGAAAAACTCGC<br>R: TAATACGACTCACTATAGGGTTCTCATGTGCCTTCTTTTT
